# Advantages of long- and short-reads sequencing for the hybrid investigation of the *Mycobacterium tuberculosis* genome

**DOI:** 10.3389/fmicb.2023.1104456

**Published:** 2023-02-02

**Authors:** Federico Di Marco, Andrea Spitaleri, Simone Battaglia, Virginia Batignani, Andrea Maurizio Cabibbe, Daniela Maria Cirillo

**Affiliations:** ^1^Emerging Bacterial Pathogens Unit, IRCCS San Raffaele Scientific Institute, Milan, Italy; ^2^Fondazione Centro San Raffaele, Milan, Italy; ^3^Università Vita Salute San Raffaele, Milan, Italy

**Keywords:** next-generation sequencing, hybrid approach, long reads, drug resistance, *Mycobacterium tuberculosis*, transmission analysis, repetitive regions

## Abstract

**Introduction:**

In the fight to limit the global spread of antibiotic resistance, computational challenges associated with sequencing technology can impact the accuracy of downstream analysis, including drug resistance identification, transmission, and genome resolution. About 10% of *Mycobacterium tuberculosis* (MTB) genome is constituted by the PE/PPE family, a GC-rich repetitive genome region. Although sequencing using short read technology is widely used, it is well recognized its limit in the PE/PPE regions due to the unambiguously mapping process onto the reference genome. The aim of this study was to compare the performances of short-reads (SRS), long-reads (LRS) and hybrid-reads (HYBR) based analysis over different common investigative tasks: genome coverage estimation, variant calling and cluster analysis, drug resistance detection and de novo assembly.

**Methods:**

For the study 13 model MTB clinical isolates were sequenced with both SRS and LRS. HYBR were produced correcting the long reads with the short reads. The fastq from the three approaches were then processed using a customized version of MTBseq for genome coverage estimation and variant calling and using two different assemblers for de novo assembly evaluation.

**Results:**

Estimation of genome coverage performances showed lower 8X breadth coverage for SRS respect to LRS and HYBR: considering the PE/PPE genes, SRS showed low results for the PE_PGRS family, while obtained acceptable coverage in PE and PPE genes; LRS and HYBR reached optimal coverages in PE/PPE genes. For variant calling HYBR showed the highest resolution, detecting the highest percentage of uniquely identified mutations compared to LRS and SRS. All three approaches agreed on the identification of two major clusters, with HYBR identifying an higher number of SNPs between the two clusters. Comparing the quality of the assemblies, HYBR and LRS obtained better results than SRS.

**Discussion:**

In conclusion, depending on the aim of the investigation, both SRS and LRS present complementary advantages and limitations implying that for a full resolution of MTB genomes, where all the mentioned analyses and both technologies are needed, the use of the HYBR approach represents a valid option and a well-rounded strategy.

## 1. Introduction

Next-generation sequencing (NGS) technologies play a fundamental role in studying microbial genomes (Köser et al., 2014). Nowadays, the whole-genome sequencing (WGS) of pathogens and viruses is routinely exploited in epidemiological outbreak analysis (Ferdinand et al., 2021), to identify and characterize bacterial pathogens and transmission chains. Recently, WGS has emerged as a powerful tool that could help in the battle of the spread of antibiotic resistance for different species (Gladstone et al., 2021). *Tuberculosis* still constitutes one of the most serious threats to human health, killing nearly 1.5 million of people per year (World Health Organization, 2021a). The higher accuracy of short-reads technology (SRS), such as Illumina, together with the use of a catalog of *Mycobacterium tuberculosis* (MTB) mutations to interpret drug resistance determinants has significantly improved the interpretation of clinical genomes (Ektefaie et al., 2021; Walker et al., 2022). The same technology has been used to investigate tuberculosis outbreaks and transmission dynamics by adopting whole-genome SNP (wgSNP) or core genome Multi-Locus Sequence Typing (cgMLST) schemes assessing genetic relatedness of MTB genomes (Kohl et al., 2014, 2018). However, short-reads technologies are not able to fully resolve hard-to-sequence regions, because has suboptimal capacity to resolve reliably large structural variations, gene duplications, or variations in repetitive regions (Modlin et al., 2021), thereby reducing coverage depth involving a lack of characterization in terms of drug resistance, virulence, and transmission analysis (Medha et al., 2021; Marin et al., 2022). Accurately resolving such regions becomes critical to close bacterial genomes, obtaining more information about virulence, evolutionary mechanisms of drug resistance, and on strain relatedness. The availability of long reads (LRS) from third-generation sequencing technologies, e.g., Oxford Nanopore Technologies (ONT) or PacBio, can improve the resolution of bacterial genomes at level of gene rearrangement, repetitive regions (proline-glutamate/proline-proline-glutamate, PE/PPE), and long insertions/deletions (InDel), usually neglected by short-read sequencing (SRS) due to their low-complex nature. Notably, ONT is a portable, robust, and low-capital-cost sequencer that could conceivably be utilized to conduct WGS analysis in a rapid manner. Recently, different bioinformatic pipelines have been developed to implement the advantages of SRS and LRS in a single unique approach (Walker et al., 2014; Wick et al., 2017, 2021b). The procedure usually involves using first SRS to make *de novo* assembly and then LRS to build the bridges between the ambiguous regions, relying mostly on the SRS steps. In this work, we aim to compare the performances of SRS, LRS, and hybrid approach on MTB clinical cluster isolates, which are resistant to first- and second-line drugs. For this purpose, we implemented the use of “hybrid reads” (HYBR), in which we first corrected the long reads with high accurate short reads, and then we used them as input for the downstream analysis, including identification of mutations, drug resistance prediction, transmission analysis, *de novo* genome assembly, and overall genome coverage. Our reverse HYBR approach outperforms the standard hybrid pipeline. Moreover, we aimed to characterize the repetitive regions of the genome, including PPE and PE genes, which are normally neglected during the SRS analysis. The outcome from this analysis indicates that PE and PPE genes, except PE_PGRS, can be included in the SRS analysis at the cost of increasing the sequencing depth. The study was performed using a subset of *M. tuberculosis* strains previously characterized in our laboratory (Mustazzolu et al., 2018; Abascal et al., 2020; Villa et al., 2021).

## 2. Materials and methods

### 2.1. Strain selection

We sequenced with the two platforms (Illumina and ONT) and perform the bioinformatic analysis with the three pipelines (SRS, LRS, and HYBR) on 13 “model” MTB clinical isolates, selected for being resistant to several drugs and in clusters (Mustazzolu et al., 2018; Abascal et al., 2020; Villa et al., 2021). The characteristics of the isolates are reported in Table 1. Our choice was based on whether LRS was accurate enough to perform standard analyses, including variant calling and cluster characterization on strains with multiple mutations conferring resistance and linked epidemiologically. The first cluster group involves preXDR strains while the second MDR strains.

**Table 1:** Isolate characteristics.

Isolate	Cluster	Year of collection	Lineage	Resistance profile
IT1708	1	2019	4.8	Pre-XDR
IT1365	1	2018	4.8	Pre-XDR
IT645	1	2017	4.8	Pre-XDR
IT1748	1	2020	4.8	Pre-XDR
IT696	1	2018	4.8	Pre-XDR
IT1508	1	2019	4.8	Pre-XDR
IT1745	1	2020	4.8	Pre-XDR
IT1313	1	2018	4.8	Pre-XDR
IT1428	1	2018	4.8	Pre-XDR
IT491	2	2009	4.3.3	MDR
MGIT84	2	2016	4.3.3	MDR
IT318	2	2010	4.3.3	MDR
IT650	2	2017	4.3.3	MDR

Lineage called using MTBseq pipeline (Kohl et al., 2018). Resistance profile according to WHO classification.

### 2.2. DNA extraction

All the strains were cultured in Middlebrook 7H9 broth in order to perform DNA extraction using Maxwell 16 Cell DNA Purification kit (Promega) and Zymo Genomic DNA Clean & Concentrator™ (D4010, D4011) kit, for Illumina and ONT sequencing, respectively.

### 2.3. Oxford nanopore technologies and illumina library preparation and sequencing

Long-reads sequencing was performed with MinION Mk1B platform (Oxford Nanopore Technologies, Oxford, United Kingdom) with a FLO-MIN106 R9.4.1 flow cell and using Rapid Barcoding Kit (SQK-RBK004) for library preparation. Short-reads sequencing was performed on NextSeq 500 and MiniSeq platforms (Illumina Inc., San Diego, CA, United States) with paired-end Nextera XT library preparation following the manufacturer’s instructions.

### 2.4. Short-reads, long-reads, and hybrid-reads data analysis

**Figure 1:**
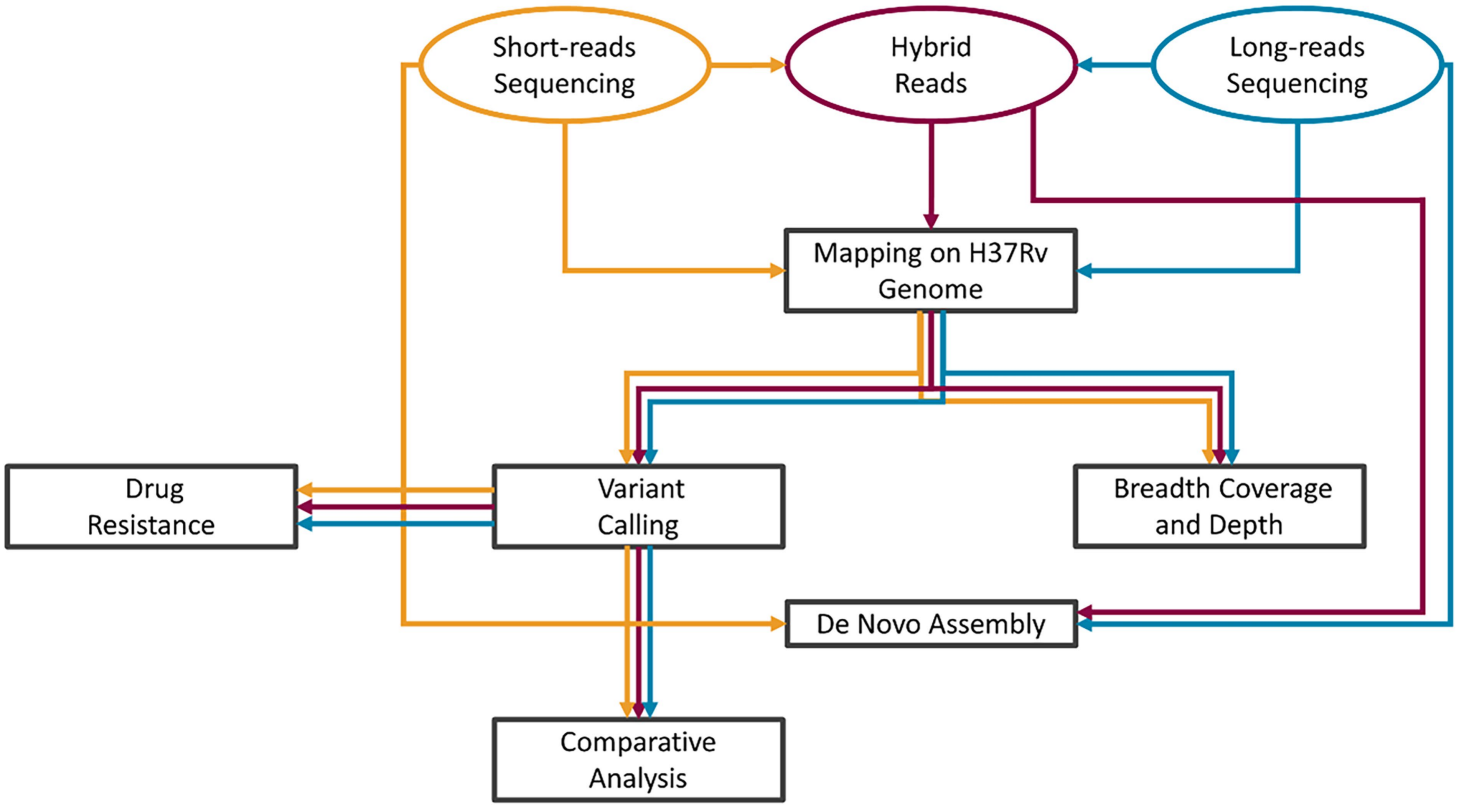
Bioinformatic analysis scheme. SRS pipeline workflow in yellow, LRS in blue, and HYBR in Red. After the acquisition of the short- and long-reads sequencing, hybrid long reads are produced. The tasks of the analyses were then performed in parallel starting from each of the three types of reads.

A graphical description of the analysis workflow is presented in Figure 1: our HYBR approach is presented in red, while the SRS and the LRS in yellow and blue, respectively. Raw fast5 files were base called using Guppy v5 to obtain LRS fastq files. The quality of the sequencing was assessed using NanoPlot v1.34.0 (de Coster et al., 2018). The HYBR approach consisted first in the correction of the long reads with short reads using Ratatosk v0.4 (Holley et al., 2021) to obtain the corrected hybrid reads. Mapping on the H37Rv genome (NCBI genome number: NC_000962.3) was performed using the BWA mem algorithm v0.7.17 (Md et al., 2019) for SRS and minimap2 algorithm v2.24 (Li, 2018) for LRS and hybrid reads. The .bam files obtained by the mapping were then processed using the MTBseq v1.0.3 (Kohl et al., 2018) pipeline starting from the TBlist step using default parameters except for *minphred20* and *minbqual* options set, respectively, at 0 and 4 and including the repetitive regions in the analysis. Distance matrices built on the unambiguously called positions in the MTBseq pipeline were used to generate transmission trees using the software GrapeTree v2.2 (Zhou et al., 2018) and samples with a distance lower than 5 SNPs were classified as closely genetically related.

H37Rv reference genome was divided into consecutive regions of 1,000 bp length and breadth coverage (defined as percentage of genome bases sequenced at a given sequencing depth) at 8x depth and was evaluated using mosdepth v0.3.1 (Pedersen and Quinlan, 2018). Median breadth coverage was plotted using Circos v0.69.8 (Krzywinski et al., 2009). One hundred and sixty-nine PE/PPE regions were also investigated. Coordinates for the repetitive regions were searched on Mycobrowser (Kapopoulou et al., 2011). Breadth coverage in the PE/PPE region was evaluated at depths 1x to 40x. Coverages between techniques were compared, performing ANOVA and *post-hoc* test with holm correction.

The detected variants using the MTBseq pipeline with a frequency higher than 10%, and at least 4 reads with a quality score higher than 20, were used for drug resistance detection, using the WHO catalog as reference (World Health Organization, 2021b): both presence of resistance-associated and *ad-interim* resistance-associated mutations were considered for this comparison.

We investigate the assembly performance between the different approaches including a fourth, namely Unicycler v0.4.4 (Wick et al., 2017), a widely used algorithm based on the short-long reads hybrid approach. The latter one exploits short and long reads simultaneously during the assembly, whereas our approach uses the short reads to first correct the long reads and then perform the assembly with Flye v2.9 (Kolmogorov et al., 2019) using the long-corrected reads. The comparison between *De Novo* assembly algorithms for LRS (Flye), only SRS (Unicycler), HYBR (Flye), and simultaneously short-long reads hybrid assembly (HYBA) (Unicycler) was assessed considering assembly metrics calculated by Quast v5.0.2 (Gurevich et al., 2013) using H37Rv as the reference genome. The considered metrics were the number of contigs, number of misassembled contigs, number of Gaps, the fraction of retrieved genes, the fraction of genome, largest alignment of the assembly, the length of the shortest contig at 50% of the total assembly length (NA50), the length of the shortest contig at the 50% of the total genome length (NG50), and number of partial genes. The results were compared between techniques performing ANOVA and *post-hoc* test with holm correction. Statistical analyses were performed using R v4.0.5 (R Core Team, 2019) and Rstudio Server 2022.02.2 (RStudio Team, 2019).

## 3. Results

### 3.1. Genome coverage

**Figure 2:**
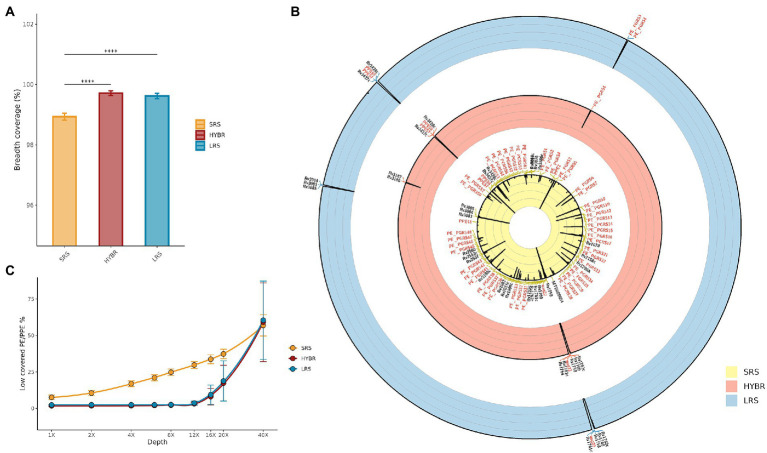
**(A)** MTB genome breadth coverage at 8x. SRS—Yellow, HYBR—Red, LRS–Blue; **(B)** Approaches genome/genes breadth coverage Circos plot at 8x. Outer to inner: Blue—LRS; Red—HYBR; Yellow—SRS. Genes with a breadth coverage lower than 90% were annotated (in red PE/PPE genes). The black line represents the 8x breadth coverage percentage at that position (0% inner–100% outer); **(C)** Number of low-covered PE/PPE genes at different levels of depths. Blue—LRS; Red—HYBR; Yellow—SRS.

In the MTBseq framework, a breadth coverage at 8X depth is assumed to be the minimum threshold to cover the whole reference genome. In Figure 2A, it is shown the fully covered genome at 8X between the three approaches, resulting different (*p* < 0.001): *post-hoc* test showed that SRS approach led to breadth coverage (98.9 ± 0.1%) lower than LRS (99.6 ± 0.1%, *p* < 0.001) and HYBR (99.7 ± 0.1%, *p* < 0.001), while LRS and HYBR performed similarly (*p* = 0.9).

Figure 2B shows the Circos plot of the breadth coverage at 8X along genome coordinates, where the black line spikes represent low-covered regions. SRS, LRS, and HYBR approach scored a low breadth coverage (<90%) in 75, 13, and 13 genes, respectively, of the whole genome. In particular, in the repetitive regions, SRS, LRS, and HYBR showed a low breadth coverage in 41, 5, and 4 genes out of 168 PE/PPE total (Figure 2B). Among the 41 PE/PPE genes with poor breadth coverage in SRS, 37 belong to the PE_PGRS family. Interestingly, HYBR presented only 1 of those genes, PE_PGRS4, with low breadth coverage, whereas LRS resulted low breadth coverage in 2 genes (PE_PGRS3 and PE_PGRS4). We studied the percentage of low-covered PE/PPE genes as function of the depth coverage (Figure 2C). SRS has an almost exponential slope by indicating that low-covered regions increase with the depths, as expected. LRS and HYBR maintain a flat trend up to 12X, afterward both approaches start to increase the number of genes low covered. All approaches present comparable low-resolution values after 40X.

**Figure 3:**
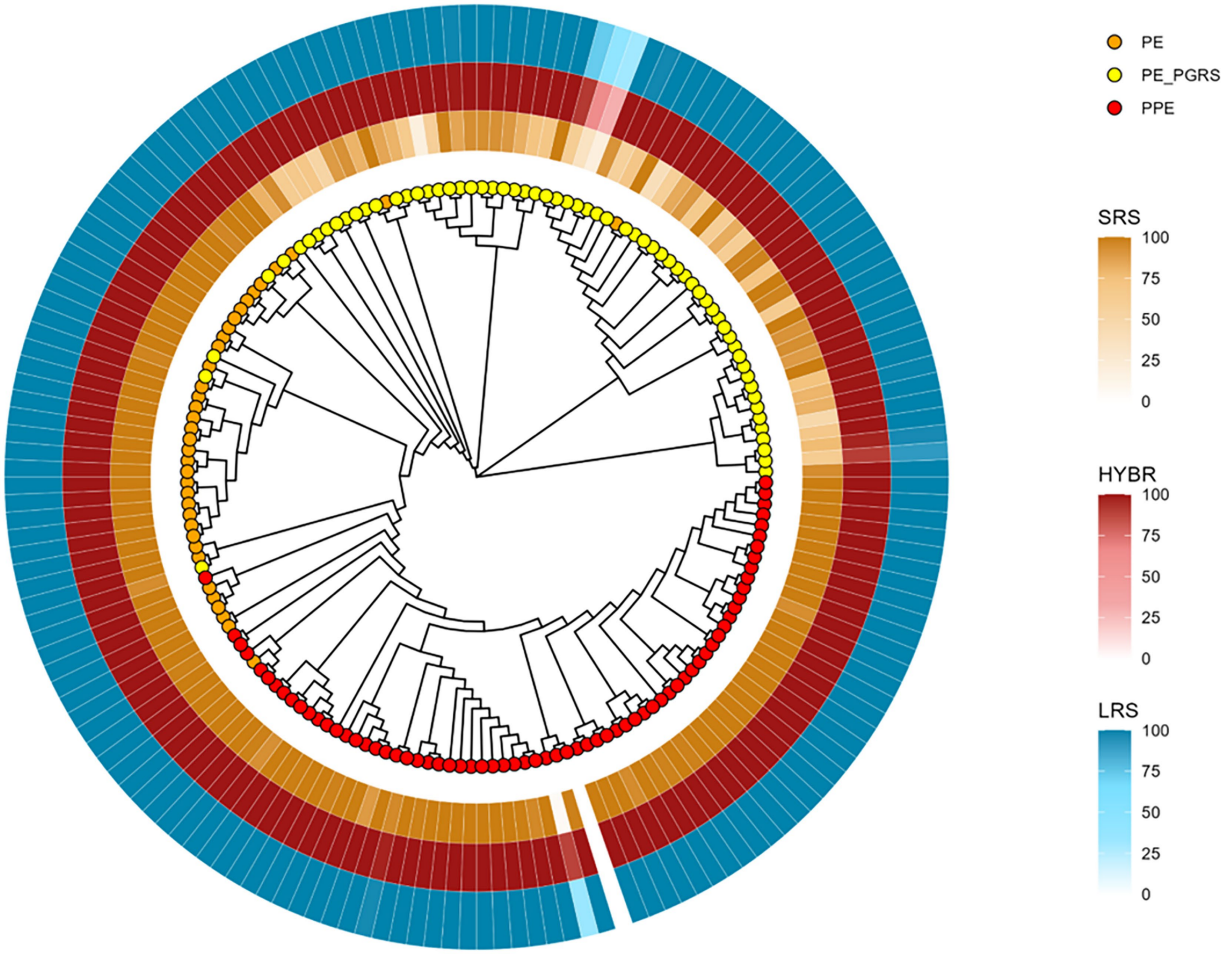
PE/PPE genes neighbor-joining tree based on multiple sequence alignment result. Orange tips: PE genes; Yellow tips: PE_PGRS genes; Red tips: PPE genes. Yellow layer: SRS 8X breadth coverage; Red layer: HYBR 8X breadth coverage; Blue layer: LRS 8X breadth coverage.

To better investigate the drops of coverage resolution, we constructed a neighbor-joining tree based only on PE/PPE reference sequences from MycoBrowser (Kapopoulou et al., 2011) to evaluate their similarities. The tree shows three different genes clades, namely PE, PPE, and PE_PGRS, respectively, orange, yellow, and red leaves (Figure 3). We then annotated the tree with the breadth coverage at 8X from our data according to the approaches (outer rings). Among the repetitive regions, the family of PE_PGRS genes shows the lowest breadth coverages in our data when using SRS, whereas they are well covered using LRS and HYBR approach.

### 3.2. Variant calling and cluster analysis

We compared the variant calls between SRS, LRS, and HYBR, using the MTBseq pipeline framework as described in the methods section. We focused our analysis to identify the single-point mutations (SNPs) present uniquely in each pipeline. The approaches showed different results (*p* < 0.001), with the *post-hoc* test showing significant differences between all the pairwise comparisons: LRS showed the lowest mean number of uniquely identified mutations (0.3 ± 0.1%), followed by SRS (1.3 ± 0.2%) and by HYBR (5.1 ± 0.4%). Considering the uniquely identified mutations not detected by the other approaches, HYBR misses 37% (36) for low coverage and 63% (62) for low frequency, SRS 68% (123) for coverage and 32% (58) for frequency, and LRS 58% (903) for coverage and 42% (651) for frequency. Among the 663 different mutations that were uniquely identified by the HYBR approach, 63% were located in the PE/PPE genes, 33% in other genes, and 4% in intergenic regions. LRS identified 46 SNPs uniquely, of which 37% located in PE/PPE genes. Finally, of 65 SNPs uniquely identified by SRS, only 23% belonged to PE/PPE genes.

**Figure 4:**
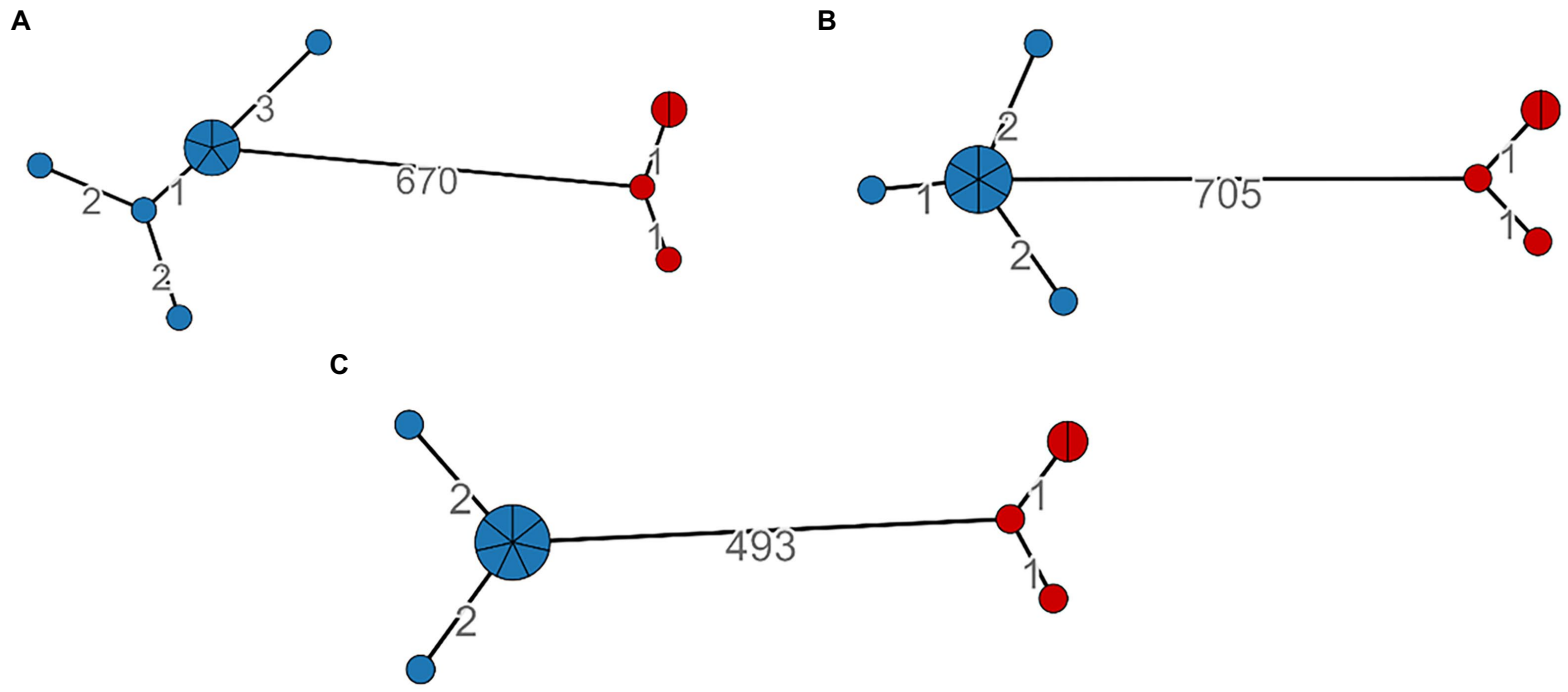
SNP-based Minimum spanning tree and clusters identification based on distance <6; Cluster 1: Blue, Cluster 2: Red. **(A)**—SRS, **(B)**—HYBR, **(C)**—LRS.

We calculated the minimum spanning tree within the MTBseq framework, and it was constructed on 499, 680, and 712 SNPs positions, respectively, for LRS, SRS, and HYBR pipelines. All three approaches agreed on the identification of two major clusters, cluster 1 and cluster 2 shown in blue and in red, respectively, (Figure 4). Cluster 2 has the same number of nodes and SNPs distance between strains (number on the edge) when analyzed with all three pipelines. Cluster 1, instead, shows a different compactness intra-cluster, namely the cluster dispersion, in all three approaches. We found that SRS identified 5 nodes (8 SNPs in total), HYBR 4 (5 SNPS) and LRS 3 nodes (4 SNPs). Although this discrepancy could reflect a different intra-cluster resolution, the strains are linked each other under the standard 5 SNPs, representing in all approaches a single chain of transmission. Finally, considering the distance between the two clusters, the HYBR approach identified a higher number of SNPs compared to SRS and LRS, due to an improved coverage of the repetitive regions (20 SNPs) and in agreement with the higher overall number of SNPs found.

### 3.3. Drug resistance

Regarding the presence of confidence-graded mutations associated with resistance to the main drugs as defined in the WHO catalog, we observed an almost perfect agreement between the pipelines to define the strains. SRS and HYBR detect identical resistance patterns, whereas LRS did not detect resistance to ethionamide in only one strain due to a low number of reads with quality higher than 20 (4/18) for the mutation “fabG1_c-15 t” associated with ethionamide and isoniazid resistance. All the approaches detected 93 high/medium confidence drug resistances associated with SNPs and 10 classified as associated with drug resistances “ad interim” (Walker et al., 2022).

### 3.4. *De novo* assembly

**Figure 5:**
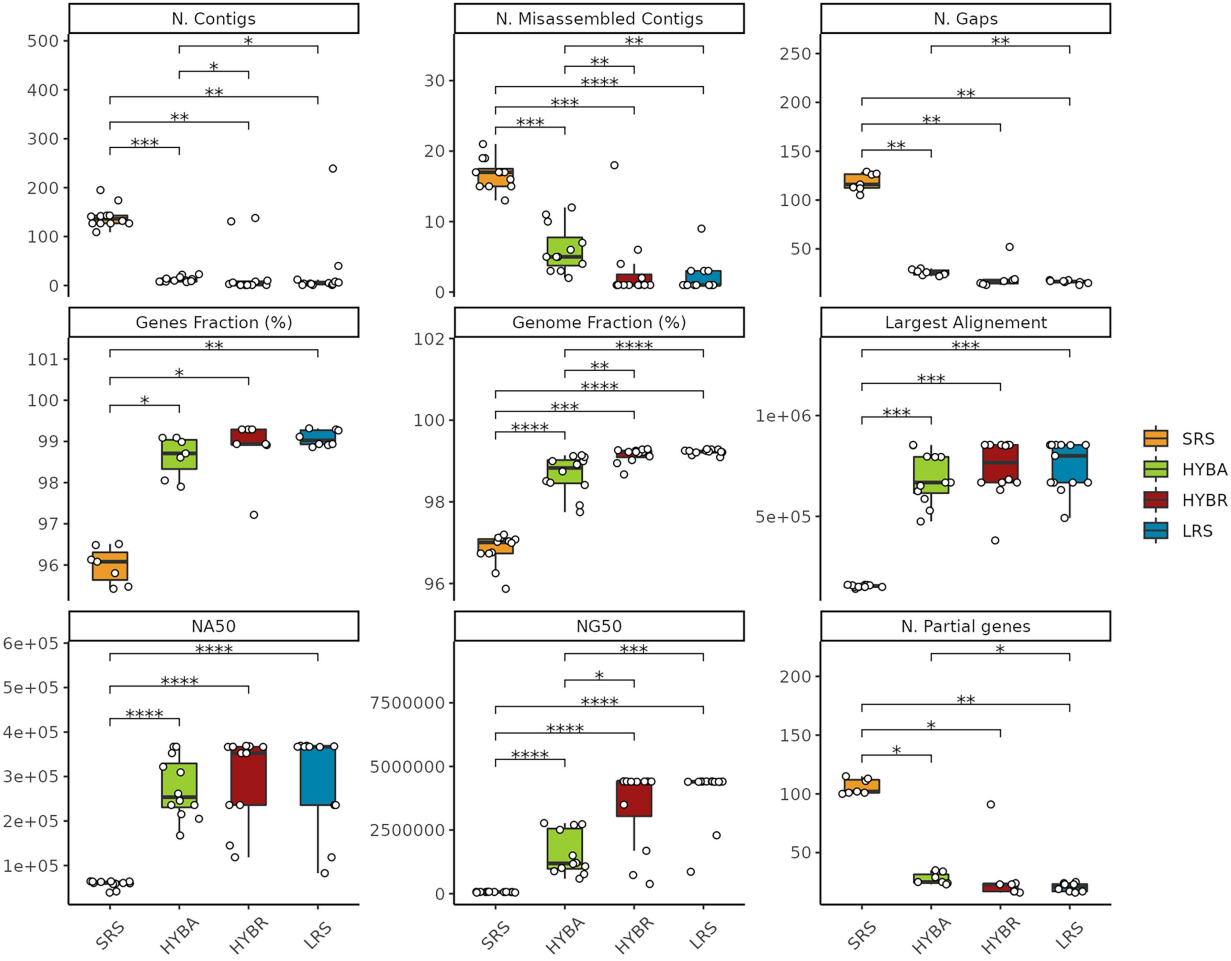
Assemblies statistics comparison. SRS in yellow, Hybrid assembly (HYBA) in green, HYBR in red, LRS in blue. *p < 0.05; **p < 0.01; ***p < 0.001; ****p < 0.0001.

We performed an assembly comparison to evaluate the importance of long reads technology. LRS and the HYBR approaches outperformed SRS and the widely-used HYBA approaches with Unicycler, in terms of number of contigs (*p* < 0.001), number of misassembled contigs (*p* < 0.001), number of gaps (*p* < 0.001), fraction of covered genome (*p* < 0.001), fraction of retrieved genes (*p* < 0.001), number of partially covered genes (*p* < 0.001), largest alignment length (*p* < 0.001), NA50 (*p* < 0.001), and NG50 (*p* < 0.001), with the SRS approach resulting the least effective for this task, as expected (Figure 5). HYBR and LRS obtained comparable results in the metrics considered. SRS obtained poor results in all the tasks, showing significant differences from the other three proposed approaches.

## 4. Discussion

The characterization of MTB strains shows different challenges associated with the aims of the genomic analysis. Several solutions were proposed over the years to optimize the analysis, with a major focus on the use of SRS (Kohl et al., 2018; Phelan et al., 2019). Differently from other prokaryotic pathogens, *MTB* shows genomic features such as the lack of mobile genetic elements (e.g., plasmids), a high GC-content, and the relevant presence of highly variable repetitive regions. All those features contribute to increase possible biases in the genomic analysis (Li and Wren, 2014; Modlin et al., 2021). Common bioinformatic pipelines usually exclude the so-called ‘biased regions’, assuming that large repeats cannot be mapped onto the reference genome unambiguously, as mappability does not depend on coverage, and the results could decrease the accuracy of the transmission analysis (Kohl et al., 2018; South et al., 2022). This compromises the possibility to identify mutations relevant for the virulence and resistance to the main drugs and to provide a comprehensive analysis of entire genomes.

The introduction of the LRS technology represents a valid alternative to the SRS approaches, because it allowed a better characterization of the MTB genome, e.g., InDel and repetitive regions. Comparing the results between SRS and LRS, different studies highlight genome regions where the SRS lacks accuracy due to limit of the technology (Modlin et al., 2021; Peker et al., 2021; Gómez-González et al., 2022; Marin et al., 2022). Although LRS approaches still present a high-error rate (~5–15%), their random nature allows to improve accuracy with higher coverage (Rhoads and Au, 2015; Athanasopoulou et al., 2021; Amoutzias et al., 2022).

In this study, we compared the performances of LRS, SRS, and our modified version of the hybrid long-short reads, HYBR, on 13 MTB strains previously described, showing MDR and preXDR patterns. We analyzed the coverage and the variant calling along the whole genome. We carried out a comparative analysis between the 3 approaches, by performing genome coverage estimation, cluster analysis, drug resistance detection, and *de novo* assembly. The results obtained showed that the implementation of the HYBR approach, which has the advantage to include the features of the long and short reads, allows a better description of the study strains in terms of genome breadth coverage and assembly compared to SRS, and variant calling and related downstream analysis compared to LRS. In fact, our hybrid approach relies on long reads first corrected by the short reads and then used them in the downstream analysis: this approach allows to adopt the newly hybrid corrected reads for all the tasks of the investigation, while usually hybrid approaches involve both LRS and SRS only for the assembly step. SRS showed several limitations in terms of coverage along the whole-genome compared to LRS and HYBR. The PE_PGRS genes regions resulted as the more problematic for SRS, although those families of repetitive genes retain an important role in terms MTB pathogenesis, and the low coverage could correspond to a not trivial loss of information in the pathogen characterization (de Maio et al., 2020). In particular, we found that PE_PGRS3 and PE_PGRS4 genes present very low coverage in all approaches. Recently, few studies characterized those specific regions in the genome, showing that they are close to each other and present a homologous sequence (percent identity of 81%) due to gene duplication, indicating that they could potentially present critical issues with every technology (Karboul et al., 2008; Phelan et al., 2016; de Maio et al., 2020). Interestingly, the remaining PE and PPE regions showed an overall acceptable coverage for SRS and as already described in other studies, the common practice of excluding those genes from the analysis, due to the high GC-content and the repetitive sequences, could be overcome by removing only the PE_PGRS genes (Modlin et al., 2021; Marin et al., 2022).

The variant calling showed how, with low depths, the high-error rate of the LRS technology masks the variants detected with the random noise produced by the basecalling step. Nevertheless, this issue could be addressed with an enhancement of the sequencing depth, differently from SRS technologies where the error is due to systematic biases (Cabibbe et al., 2020). The HYBR approach outperformed both SRS and LRS, the latter missing few mutations due to coverage issues in those regions. The hybrid reads approach requires a good sequencing depth from LRS otherwise it will inherit the same issues of the parental LRS in terms of signal/noise ratio, especially in those regions where SRS correction does not perform optimally. In fact, most of the undetected mutations were due to frequency threshold (75%), especially in those regions not well covered by SRS. Nevertheless, considering the repetitive regions, this result indicates that HYBR approach can reveal a great number of mutations compared to SRS and LRS, due a better coverage.

In the *de novo* assembly evaluation, the three approaches were compared among each other and to the widely used hybrid assembler Unicycler (de Maio et al., 2019; Wick et al., 2021a). As stated by the developer, the hybrid assembly executed by Unicycler corresponds to a “short-read-first” approach in which the short reads assembly graph is scaffolded to completion by the long reads (Wick et al., 2017). This approach was proposed with the assumption that LRS presents low depth and accuracy. The improvement of the ONT technology claiming to lower error rate at 1% with the introduction of the new 10.x flow cell chemistry, allowed to rely on the opposite “long-reads-first” approaches as Trycicler (Wick et al., 2021b). In the current comparison, the LRS still relies on the previous technology presenting low depth and accuracy. Nevertheless, the HYBR and the LRS showed the best results, confirming Flye as one of the best-performing assemblers for long reads (Wick et al., 2021a). Interestingly, in our dataset, the hybrid assembler Unicycler performed poorly than Flye, especially considering the NG50 metrics (the length of the shortest contig at 50% of the total genome length), presenting a mean of 1.6 ± 0.3 Mb, lower than the HYBR with 4.3 ± 0.1 Mb (*p* = 0.02), indicating that our HYBR can better assembly the genomes. As expected for this task, SRS performed very poorly emphasizing its inadequacy for the *de novo* assembly.

This study presents some limitations: the limited number of samples considered for the analysis despite the deep investigation conducted on each genome and the adoption of the 9.x flow cells technology for LRS bearing a higher error rate compared to the new 10.x as the latter was not available at the time of the study.

This study outlines the strengths and the weaknesses of three approaches. The repetitive regions of the PE_PRGS genes represent a source of blind spots for the SRS, while the remaining PE/PPE regions, usually neglected as well, could be safely included in the analysis, showing good coverage. The LRS shows issues in terms of signal-to-noise ratio but still can correctly identify genetically closed strains and drug resistance-associated mutations, and the increase of sequencing depth enables usually to fix the issue. The HYBR approach overcomes the limitations of both SRS and LRS, showing the best results in all the considered tasks. Although hybrid reads approach suffers from the relative higher cost compared to the single sequencing run of SRS and LRS, it could offer the advantage to better evaluate problematic regions in variant calling, where LRS presents critical issue, and in *de novo* assembly, where SRS cannot compete with LRS.

**Figure 6:**
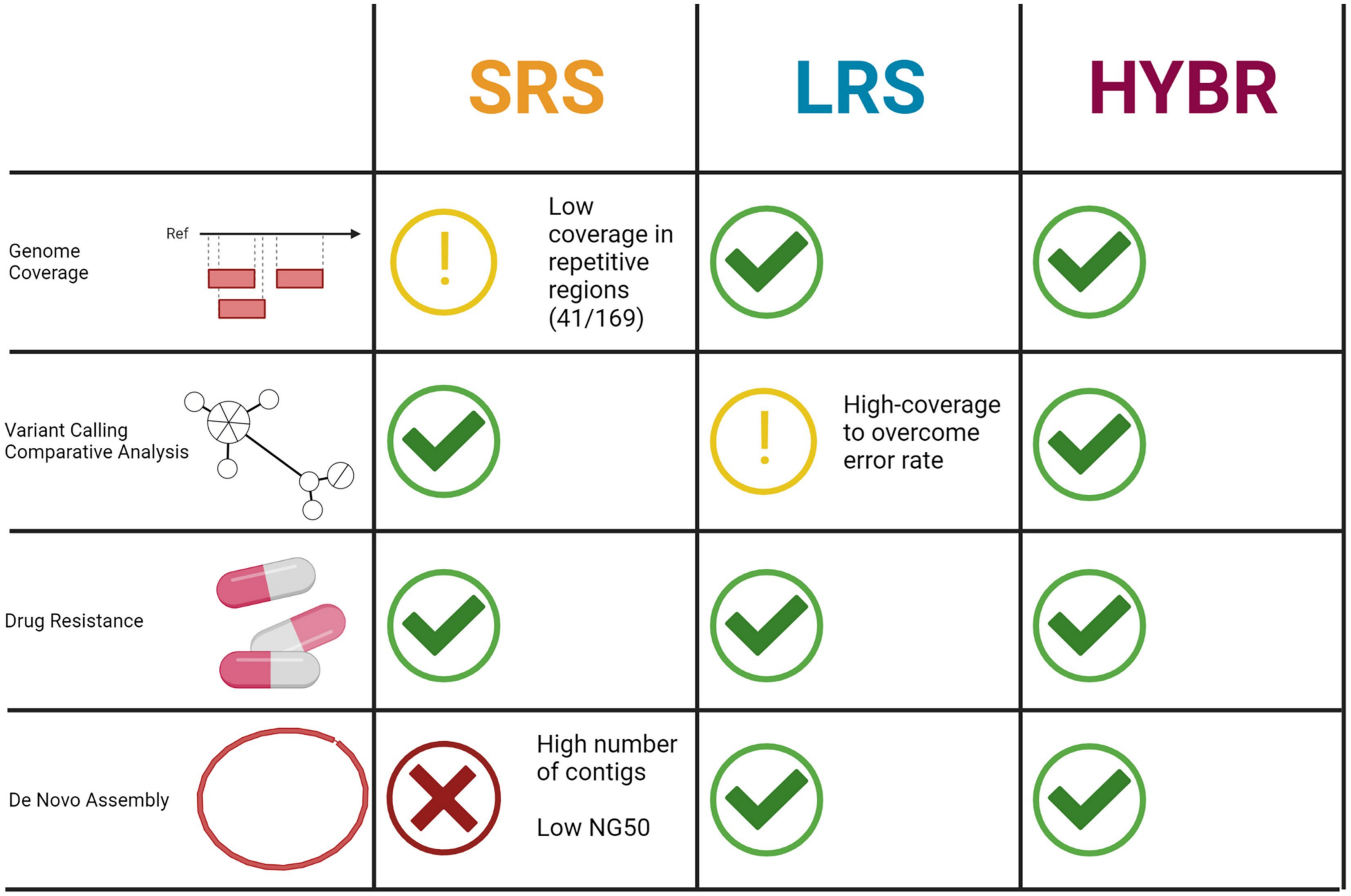
Tasks performance comparison between three approaches. Created with BioRender.com.

In conclusion, depending on the aim of the investigation, both SRS and LRS present complementary advantages and limitations implying that for a full resolution of MTB genomes, where all the mentioned analyses and both technologies are needed, the use of the hybrid reads approach represents a valid option and a well-rounded strategy (Figure 6).

## Data availability statement

The datasets presented in this study can be found in online repositories. The names of the repository/repositories and accession number(s) can be found at: https://www.ncbi.nlm.nih.gov/, PRJNA903660. The codes used for the analysis presented in the study are deposited in the Github repository, accessible at: https://github.com/Allen13x/MTB_LRSvsSRS.

## Author contributions

FDM, AS, SB, AMC, and DMC conceived and supervised the study. SB and VB performed the sequencing. FDM and AS performed the bioinformatics analysis. FDM and AS wrote the draft manuscript. FDM, AS, AMC, and DMC revisioned the draft manuscript. All authors contributed to the article and approved the submitted version.

## Funding

This study was partially supported by the 2nd ERANet-LAC Transnational Joint Call on Research and Innovation (grant: TRANS-TB-TRANS PER-2012-ELAC2015/T08-0664).

## Conflict of interest

The authors declare that the research was conducted in the absence of any commercial or financial relationships that could be construed as a potential conflict of interest.

## Publisher’s note

All claims expressed in this article are solely those of the authors and do not necessarily represent those of their affiliated organizations, or those of the publisher, the editors and the reviewers. Any product that may be evaluated in this article, or claim that may be made by its manufacturer, is not guaranteed or endorsed by the publisher.
